# Urohidrosis as an overlooked cooling mechanism in long-legged birds

**DOI:** 10.1038/s41598-021-99296-8

**Published:** 2021-10-08

**Authors:** Julián Cabello-Vergel, Andrea Soriano-Redondo, Auxiliadora Villegas, José A. Masero, Juan M. Sánchez Guzmán, Jorge S. Gutiérrez

**Affiliations:** 1 Conservation Biology Research Group, Faculty of Sciences, University of Extremadura, 06006 Badajoz, Spain; 2 Centro de Investigação Em Biodiversidade E Recursos Genéticos, Laboratório Associado, Universidade Do Porto, Rua Padre Armando Quintas 7, 4485-661 Vairão, Portugal; 3 Centro de Investigação Em Biodiversidade E Recursos Genéticos, Laboratório Associado, Instituto Superior de Agronomia, Universidade de Lisboa, Tapada da Ajuda, 1349-017 Lisbon, Portugal; 4 Ecology in the Anthropocene, Associated Unit CSIC-UEX, Faculty of Sciences, University of Extremadura, Badajoz, Spain

**Keywords:** Ecology, Behavioural ecology, Climate-change ecology, Ecology, Behavioural ecology, Climate-change ecology

## Abstract

Behavioural thermoregulation could buffer the impacts of climate warming on vertebrates. Specifically, the wetting of body surfaces and the resulting evaporation of body fluids serves as a cooling mechanism in a number of vertebrates coping with heat. Storks (Ciconiidae) frequently excrete onto their legs to prevent overheating, a phenomenon known as urohidrosis. Despite the increasingly recognised role of bare and highly vascularised body parts in heat exchange, the ecological and evolutionary determinants of urohidrosis have been largely ignored. We combine urohidrosis data from a scientifically curated media repository with microclimate and ecological data to investigate the determinants of urohidrosis in all extant stork species. Our phylogenetic generalised linear mixed models show that high temperature, humidity and solar radiation, and low wind speed, promote the use of urohidrosis across species. Moreover, species that typically forage in open landscapes exhibit a more pronounced use of urohidrosis than those mainly foraging in waterbodies. Substantial interspecific variation in temperature thresholds for urohidrosis prevalence points to different species vulnerabilities to high temperatures. This integrated approach that uses online data sources and methods to model microclimates should provide insight into animal thermoregulation and improve our capacity to make accurate predictions of climate change’s impact on biodiversity.

“Because urohidrosis is probably rather complicated, from a physiological and genetic standpoint, it may be valuable in assessing taxonomic relationships. Clearly more work needs to be done on this problem.” Hancock et al. (1992, p. 145)

## Introduction

Endotherms can deal with heat stress through a series of physiological and behavioural responses in order to maintain their thermal balance. The first response is likely to be behavioural, such as the selection of cooler microclimates or the use of heat-dissipating behaviours^1–4^; if those responses are not sufficient, they must perform rapid physiological adjustments^5^. Although not without cost^6^, behavioural responses can maximize or postpone the beginning of costly physiological cooling mechanisms, thus contributing to energy and water conservation^7,8^. According to the ‘heat dissipation limit theory’—which posits that heat generated during metabolism limits energy intake and, ultimately, reproductive output in endotherms^9^—behavioural thermoregulation could maximize heat dissipation capacity allowing individuals to allocate energy to other activities different than cooling, such as foraging, mating or brooding^6^. In accordance with this theory, recent studies with free-ranging birds have experimentally supported that constraints on heat dissipation rate could be a key mediator of life-history trade-offs^10–12^. Indeed, the potential for behavioural thermoregulation to buffer endotherms against climate warming has been recognized^3,13^, and studies on patterns of heat dissipation behaviours have gained attention in recent years^2–4,14–18^. However, studies that incorporate behavioural responses when modelling the impacts of climate warming are still rare (but see^19–23^), and the opportunity costs and the response of endotherms to climate warming have been largely overlooked^6^.

Birds are one of the most vulnerable groups against global warming owing to their typically diurnal habits, small body size, relatively high metabolic rates and limited use of thermal refuges^24^. Panting (sometimes accompanied by gular fluttering) is usually the main thermoregulatory response in birds when environmental temperature exceeds their upper thermoneutral zone limit^25,26^. Yet panting is costly because it requires a large expenditure of water and can cause changes in blood chemistry^27^. Therefore, birds have evolved a series of heat dissipation behaviours that allow them to postpone the beginning of panting to higher temperatures and/or maximize its cooling capacity (e.g.^2,4,8^). These include postural adjustments, ptilomotor responses, bathing or watering behaviours, shade seeking, or the reduction of activity levels^1,3,21,28,29^.

**Figure 1:**
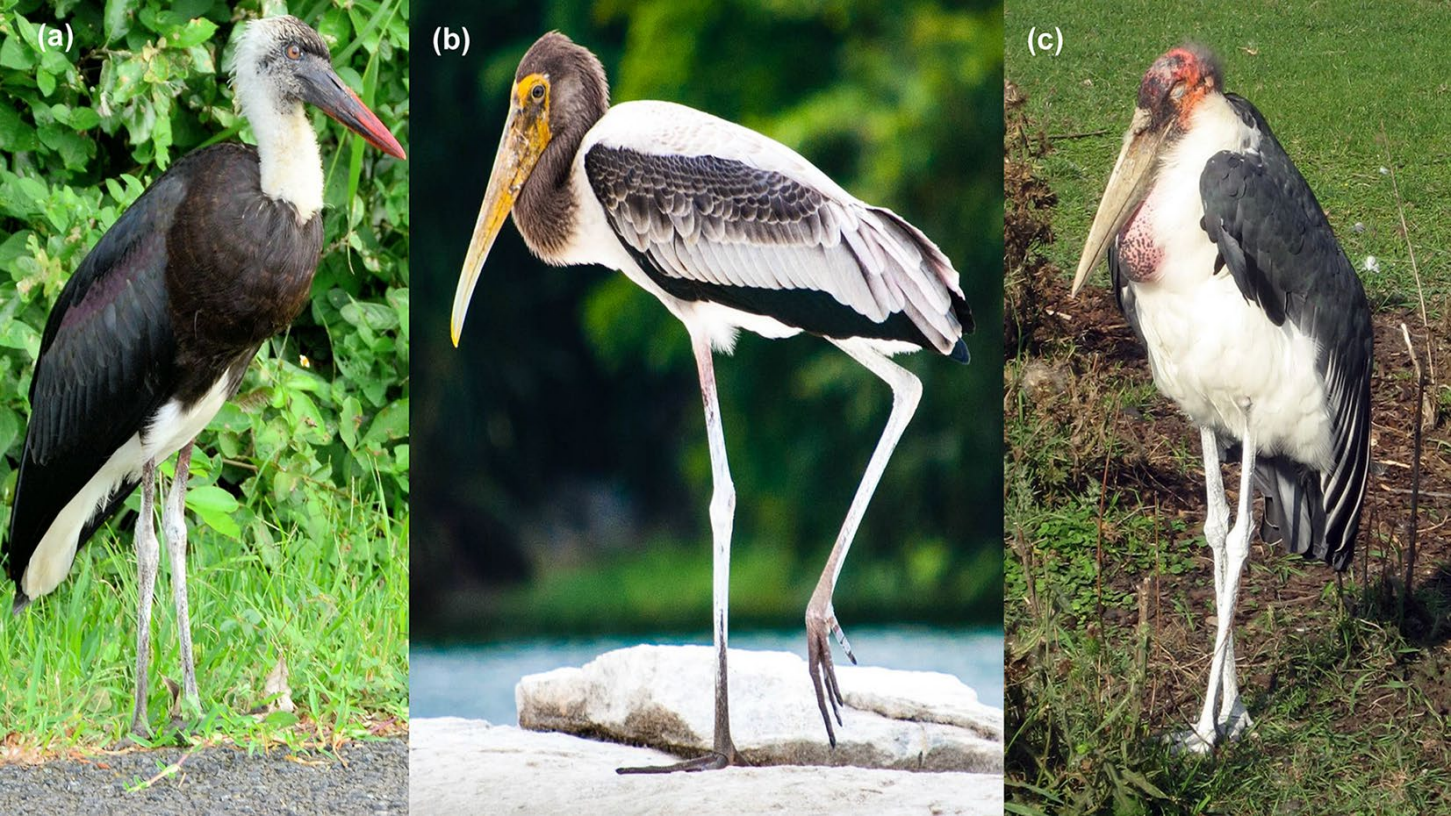
Examples of urohidrosis in different stork species: (**a**) woolly-necked *Ciconia episcopus*, (**b**) painted *Mycteria leucocephala* and (**c**) marabou *Leptoptilos crumenifer* storks. In the first two, the whitish residue produced by urohidrosis covers partially the limbs with feet showing its natural darker colour. In the case of marabou, excreta covers completely the limbs, including the feet. Credit for images: woolly-necked stork – Ossewa (distributed under CC BY-SA 4.0 license; https://creativecommons.org/licenses/by-sa/4.0/), painted stork – Unni Hariharan (CC BY-SA 4.0; https://creativecommons.org/licenses/by-sa/4.0/) and marabou – Dezidor (CC BY 3.0; https://creativecommons.org/licenses/by/3.0/).

At the same time, studies on avian thermoregulation using infrared thermal imaging have demonstrated that unfeathered and well-vascularized appendages like legs or bills can act as ‘thermal radiators’^30–32^. This could be particularly important for storks (Ciconiidae), as they are long-legged birds that typically inhabit warm, open habitats where heat stress can be problematic^33^. In fact, storks can regulate their blood flow to the legs depending on ambient temperature, promoting vasoconstriction under cold and vasodilation under heat exposition^34,35^. Notably, storks can deliberately excrete onto their legs when exposed to increasing environmental temperatures, a phenomenon known as urohidrosis^34^ (Fig. 1). When overheated, storks repeatedly direct liquid excreta toward their legs (only one leg hit at a time), which usually evaporates before reaching the toes^34^. Analogously, the wetting of other body surfaces and the resulting evaporation of body fluids (saliva, mucous, urine or wet faeces) serves as a cooling mechanism in a number of terrestrial, and some marine, vertebrates coping with heat stress^36–39^.

Urohidrosis is relatively rare in birds because it requires regular access to drinking water, but New World vultures, condors, storks, gannets, and boobies engage in this behaviour^34,40–45^. Since Kahl’s^34^ pioneer work, however, little attention has been paid to the role of urohidrosis for thermoregulation and its underlying mechanisms remain largely unexplored. Kahl reported that urohidrosis is mainly determined by maximum ambient temperature in wood storks *Mycteria americana*; however other environmental variables that also influence heat balance, such as wind speed,solar radiation and humidity, might play a role too. Kahl hypothesized that this behaviour might be more common in tropical species than in temperate species as the latter are less often exposed to heat. He thus proposed urohidrosis to be an adaptive response to life in warm and open habitats with regular access to water. This view is supported by the fact that storks usually, but not always, inhabit landscapes where water sources are fairly accessible^33^. Along this line, a recent study^46^ has shown that regularly drinking species have greater cooling capacities and heat tolerance limits than non-drinking species. Hence, interspecific variation in the use of urohidrosis could arise as a function of their dependency on waterbodies.

Here, we combine urohidrosis data from the largest scientifically curated archive of natural history media (photos and videos) with historical microclimate data to investigate the ecological and evolutionary determinants of urohidrosis in all extant stork species. We investigate whether local environmental conditions, latitude, foraging habits, plumage colour, and size (body mass and tarsus length) influence the use of urohidrosis across species. We predict that higher environmental temperatures and a lower dependency on waterbodies promote the use of urohidrosis in order to maintain the heat-water balance. Because of the potentially higher rates of ‘dry’ (non-respiratory) heat loss in species with longer legs^47,48^, we therefore predict that for species with similar ecological requirements, those with longer legs will use urohidrosis relatively more often to increase heat loss. Likewise, heavier species could exhibit a more pronounced use of urohidrosis due to their relatively larger body water reserves. Finally, we expect darker species to engage in this behaviour more often than lighter ones due to the higher radiation absorptivity of dark plumages^49^.

Lastly, to assess storks’ vulnerability to high temperatures, we also determined the air temperature at which urohidrosis occurred in 50% of instances for each species separately. Such temperature thresholds are useful in investigating the ecological factors shaping thermal physiological trade-offs, and for understanding ecological and evolutionary determinants of species persistence in hot environments^4^.

## Materials and methods

### Data collection

#### Urohidrosis data

**Figure 2:**
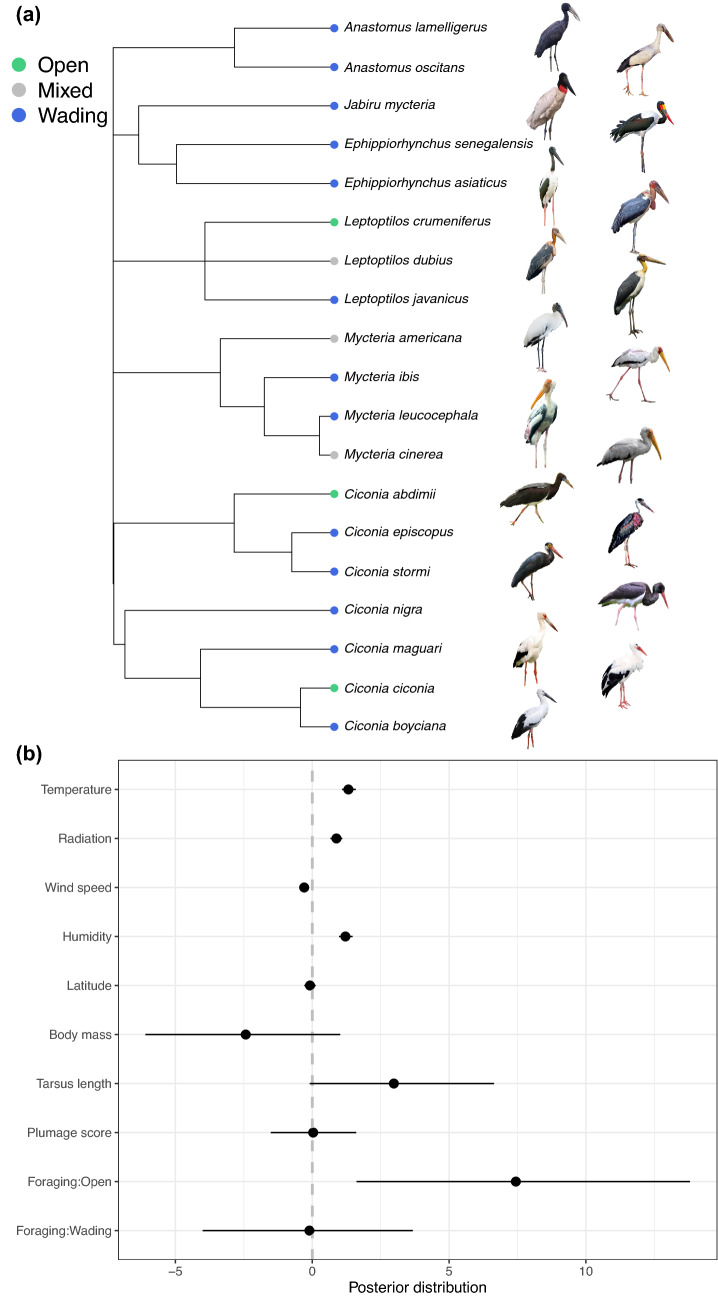
(**a**) Phylogenetic tree showing the foraging habits of stork species analyzed in this study. (**b**) Posterior distributions (with 95% CI) of predictors estimates from phylogenetic generalized mixed models. Significance is indicated by estimates not crossing zero. Reference level for foraging habit is “mixed”. Species pictures were taken from photographs distributed under CC BY licenses. Credits for pictures: African openbill and maguari stork – Lip Kee Yap (distributed under CC BY 2.0; https://creativecommons.org/licenses/by-sa/2.0/), Asian openbill – Shino J Koottanad (CC BY-SA 4.0; ), jabiru – Gmmv1980 (CC BY-SA 4.0; ), saddle-billed, yellow-billed and black storks – Bernard Dupont (CC BY-SA 2.0; ), black-necked stork – JJ Harrison (CC BY-SA 4.0; ), marabou and Abdim’s stork – Charles J Sharp (CC BY-SA 4.0; ), greater adjutant – Yathin S Krishnappa (CC BY 3.0; ), lesser adjutant – Irothu (CC BY-SA 4.0; ), wood stork – Kaldari (CC0 1.0; ), painted stork – Manvendra Banghi (CC BY-SA 2.0; ), milky stork – Gerifalte Del Sabana (CC BY-SA 4.0; ), woolly-necked stork – Shantanu Kuveshkar (CC BY-SA 4.0; ), storm’s stork – Mike Prince (CC BY 2.0; ), white stork – André Karwath (CC BY-SA 2.5; ), oriental stork – Alpsdake (CC BY-SA 4.0).

We searched for videos and photos of all 19 extant stork species (Fig. 2a) on the Macaulay Library repository (https://www.macaulaylibrary.org; the world’s largest scientifically curated archive of natural history media). Since 2020, this online repository also integrates the Internet Bird Collection (https://www.hbw.com/ibc), another repository to videos, photos, and audio recordings from the worldwide community of birdwatchers.

We examined all available images and videos of storks. Of these, we selected 6,112 Macaulay files in which we could determine for each focal individual the presence or absence of urohidrosis with confidence (Supplementary Information Material S1). We recorded the presence/absence of the chalky, whitish residue produced by urohidrosis in each individual (Supplementary Fig. S1). We only selected for analyses those photographs or video recordings in which legs were well visible (either in individuals standing or in flight). We discarded files with poor lighting and/or low sharpness. The number of files varied widely across species (range = 38–1534; see Supplementary Information Material), most likely due either to the rarity or conspicuity of the species in question. We also recorded the date and geographic coordinates in order to associate urohidrosis to environmental conditions (see below).

#### Morphological and ecological data

For each species, we extracted from the literature data on mean tarsus length (mm; a proxy of leg length)^43^ and mean body mass (g)^50^. Depending on their dependency on waterbodies for foraging activities^50^, we classified stork species as: ‘wading’ (species that mainly feed on fishes and aquatic invertebrates in waterbodies), ‘open’ (species that mainly feed on terrestrial invertebrates, micromammals and carrion in dry landscapes, such as grassland and savanna habitats) or ‘mixed’ species (those with mixed diets that forage both on land and in waterbodies).

#### Plumage scoring

Following Brooke^51^, we scored the plumage colour of the upperparts of each stork species by using colour plates available in *Birds of the World*^33^. We focused on the upperparts because they are exposed to solar radiation most of the time (either when foraging, resting, flying or breeding). Head, neck, back, wing coverts, primaries and secondaries were scored as 0 when light or as 1 if dark coloured. A total score was obtained by summing up scores in each part, so it ranged from 0 (totally light upperparts) to 6 (totally dark upperparts).

#### Microclimatic variables

We used the *microclima* R package^52^ to obtain microclimatic data for each media file considered in this study. The function ‘hourlyNCEP’ allows to obtain historical hourly values of several microclimatic variables (maximum air temperature, humidity, pressure, wind speed, wind direction, emissivity, cloud cover and various radiation parameters). We used this function to extract for each focal stork historical microclimate data for the observation date as well as for the previous day, as the presence/absence of urohidrosis can be defined as a ‘point event’ (i.e., the behaviour has no duration or its duration is unknown). We considered a conservative time span of two days from the observation as we could not know if the urohidrosis event occurred the day of the observation or earlier. Although urohidrosis residue marks might last even longer, we only included recent events as the inclusion of older, faded marks could have overestimated the use of urohidrosis and thus bias our results (see Discussion).

For the analyses, we selected the following microclimatic variables: mean maximum air temperature (hereafter temperature, $^\circ$C), mean solar radiation (radiation, MJ m^−2^ h^−1^), mean wind speed (wind speed, m s^−1^) and mean specific air humidity (humidity, kg kg^−1^). All of these were calculated as the average between the values of the day of the observation and the previous day.

### Statistical analyses

#### Interspecific analyses

We modelled the probability of urohidrosis as a function of microclimatic variables and ecological/morphological traits by fitting Bayesian phylogenetic linear mixed models using the package *MCMCglmm*^53^. We set urohidrosis as a binary (presence/absence) response variable, with temperature, radiation, wind speed, humidity, absolute latitude, mean body mass, mean tarsus length, plumage score, and foraging habit as predictor variables. All continuous variables were scaled in order to facilitate results interpretation. We included media file ID as a random effect to account for possible pseudoreplication derived from the observation of multiple individuals in a given file. To control for phylogenetic effects, we included a consensus tree as random effect. To do this, we first obtained 10,000 trees with different topologies from the Bird Tree project^54^ for the 19 extant stork species, using ‘Hackett All Species’ as backbone. We then derived an ultrametric and rooted consensus tree using the package *phytools*^55^. For all analyses, we used weakly informative priors for random effects and ran MCMC chains for 5,000,000 iterations with a burnin of 100,000. We did not detect signs of collinearity between predictors, with all showing variance inflation factors (VIF) values lower than 5. The effect of a given predictor was considered significant if zero was not included in the 95% confidence interval (CI). *P*-values were calculated automatically by the function based on this assumption and named ‘pMCMC’ in the package *MCMCglmm*^53^.

The estimated phylogenetic variance in the model was used to calculate heritability (*h*^2^), a measure of phylogenetic signal equivalent to lambda in phylogenetic generalized least squares models^56^. *h*^2^ ranges between 0 and 1, with values close to 1 indicating a strong phylogenetic signal in the data and values close to 0 suggesting a negligible phylogenetic signal.

#### Intraspecific analyses

We constructed similar generalized linear mixed models for each species separately, with urohidrosis as the response variable and microclimatic variables (temperature, radiation, wind speed, and humidity) and absolute latitude as predictors. Again, media file ID was included as a random effect. We used the package *lme4*^57^ to construct mixed effects logistic regression models with a binomial error and logit-link function (fitted by maximum likelihood)^58^. First, we included all predictor variables in a global model and then performed model selection using the ‘dredge’ function in *MuMIn* package^59^. We compared among models using an AICc framework, choosing the model with the lowest AICc score as the best supported model. As any of the best supported models had a model weight (w_*i*_) greater than 0.90, we used (‘full’) model averaging (using models with ΔAICc < 2) to identify the most important predictor variables^60,61^. We checked each model for overdispersion (values less than 1.5 indicating no issues) and collinearity (all VIF < 5).

When temperature emerged as a significant predictor, we ran separate models to determine the ambient temperature threshold at which urohidrosis is present in the 50% of the observations. Following Smit et al.^4^, this threshold was determined by dividing intercept’s absolute value by beta’s absolute value.

However, for some species we could not find enough media files that covered a range of latitude or climatic conditions (see Supplementary Table S1) representative of the environmental gradient that they typically experience throughout the year; that was the case for storm’s stork *Ciconia stormi* and milky stork *Mycteria cinerea*. Thus, we discarded both species from intraspecific analyses. Similarly, due to model convergence issues, we could not perform analyses for species in which urohidrosis in our dataset is anecdotal; that was the case for saddle-billed *Ephippiorhynchus senegalensis* (instances of urohidrosis: n = 13), black-necked *Ephippiorhynchus asiaticus* (n = 12), maguari *Ciconia maguari* (n = 8), black *Ciconia nigra* (n = 2) and oriental *Ciconia boyciana* (no records of urohidrosis) storks. Therefore, we finally ran intraspecific models for the remaining 12 species for which enough observations of urohidrosis were available over a wide range of latitude and climatic conditions.

All analyses were performed in R^62^. Results are shown as means ± 95% CI.

## Results

### Interspecific comparisons

All microclimatic variables emerged as significant predictors (Fig. 2b; Supplementary Table S2). Urohidrosis was positively associated with temperature (*β* = 1.321*,* CI = 1.091, 1.591), radiation (*β* = 0.884*,* CI = 0.665, 1.097) and humidity (*β* = 1.213, CI = 0.978, 1.474), while was negatively associated with wind speed (*β* = −0.294, CI = −0.468, −0.134). Moreover, foraging habit had a significant effect, with open foraging having a significant positive influence on urohidrosis (*β* = 7.441, CI = 1.618, 13.804) (Fig. 2b). On the other hand, latitude (*β* = −0.0818, CI = −0.289, 0.124), plumage score (*β* = 0.034, CI = −1.516, 1.606), body mass (*β* = −2.430, CI = −6.097, 1.023) and tarsus length (*β* = 2.982, CI = −0.091, 6.644) were not significant predictors of urohidrosis, albeit tarsus length had a marginally significant effect (only 7.35% of its posterior distribution overlapping zero) (Fig. 2b).

Overall, data showed a low phylogenetic signal (*h*^2^ = 0.059).

### Intraspecific comparisons

**Figure 3:**
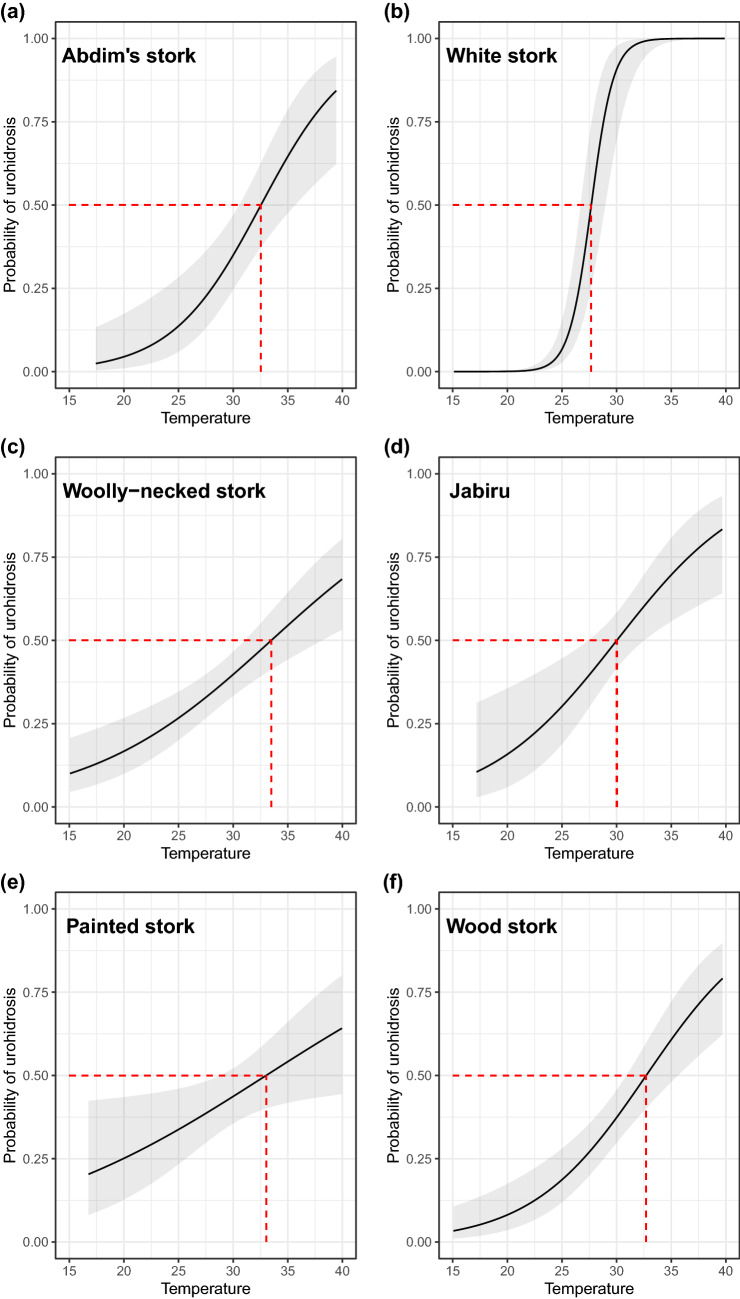
Probability of urohidrosis use in response to air temperature for (**a**) Abdim’s stork *Ciconia abdimii,* (**b**) white stork *Ciconia ciconia*, (**c**) woolly-necked stork *Ciconia episcopus*, (**d**) jabiru *Jabiru mycteria*, (**e**) painted stork *Mycteria leucocephala* and (**f**) wood stork *Mycteria americana*. Dashed red lines indicate the air temperature at which urohidrosis occurred in 50% of instances.

Model-averaged values (ΔAICc < 2) showed that determinants of urohidrosis varied across the 12 stork species analyzed (Supplementary Table S3). As expected, the most significant predictor across species was temperature, having a positive effect on urohidrosis in six of the 12 species (Supplementary Table S3). The temperature at which 50% of the individuals presented urohidrosis varied across species, ranging from 27.68 $^\circ$C in the white stork *Ciconia ciconia* to 33.49 $^\circ$C in the woolly-necked stork *Ciconia episcopus* (Fig. 3).

Moreover, the use of urohidrosis increased with radiation in the wood stork (Supplementary Table S3), and with humidity in the white stork and in the lesser adjutant *Leptoptilos javanicus* (Supplementary Table S3). Likewise, latitude was positively associated with urohidrosis in the greater adjutant *Leptoptilos dubius* and painted stork *Mycteria leucocephala*, but showed a negative effect in the white stork and the jabiru *Jabiru mycteria* (Supplementary Table S3). Nevertheless, the use of urohidrosis was not influenced by any of the studied variables in the African *Anastomus lamelligerus* and Asian *Anastomus oscitans* openbills, the marabou *Leptoptilos crumenifer* and the yellow-billed stork *Mycteria ibis* (Supplementary Table S3).

Finally, wind speed was not a significant predictor of urohidrosis use in any of the species analysed (Supplementary Table S3).

## Discussion

Many endotherms inhabiting hot, open environments regularly cope with operative temperatures that approach or exceed their body temperature, thus facing a trade-off between dehydration avoidance and resistance to hyperthermia^46^. In storks and other avian clades, urohidrosis is believed to be an important thermoregulatory behavioural response to avoid hyperthermia^34^. In this study, we examined the factors driving the use of urohidrosis by storks and its potential implications for thermoregulation in hot environments. After controlling for latitudinal and evolutionary patterns, we found that high environmental temperature, solar radiation and air humidity, as well as low wind speed, promote the use of urohidrosis across species. This finding confirms earlier experiments with wood storks showing that urohidrosis is mainly determined by maximum ambient temperature^34^, but also shows that other environmental variables jointly determine the use of this cooling mechanism. This is no surprise as three of these variables (temperature, radiation and wind speed) make up ‘operative temperatures’ that affect an animal’s heat balance (reviewed in^63^). Besides, increased air humidity can constrain evaporative heat loss through panting or through the skin^64,65^ and may force birds to rely more on urohidrosis to increase total heat dissipation. As predicted, our interspecific analyses also showed that species with a lower dependency on waterbodies (i.e. ‘open’ species typically foraging on land) exhibited a more pronounced use of urohidrosis than those mainly foraging in waterbodies (i.e. ‘wading’ or ‘mixed’ species). Open-foraging species with more limited access to waterbodies rely more on urohidrosis, presumably to maximize heat loss through evaporative cooling. Heat-load problems will occur more frequently in exposed places where heat gain is high (high temperatures and radiation), especially if the heat-loss potential is low (low winds, high humidity) (see^66^). On the other hand, wading species could potentially lose all of their heat production if standing in water, as the thermal conductivity of water is 25 times that of air^67^. Notably, Fitzpatrick et al.^68^ modelled the thermoregulatory implications of wading in whooping cranes *Grus americana* and showed that the ‘upper critical temperature’ increased substantially when legs were submerged in water. Wading storks could therefore benefit from the higher convective heat transfer of water, increasing net heat loss and diminishing heat stress while foraging. It is possible that ‘wading' or ‘mixed’ species rely on urohidrosis while exposed to more stressful thermal conditions, e.g. during reproduction, when parents must balance thermoregulation against breeding activities^8^. Together, our results support the notion that urohidrosis is an adaptive response for life in open and warm habitats.

However, dark- and light-pigmented storks did not differ in their use of urohidrosis. Although darker colours absorb more radiation than lighter colours^49,69^, evidence that darker birds are limited from occupying environments with high temperatures is mixed^49,70^. Melanin-rich, dark feathers and skins are more resistant to abrasion and can protect birds against ultraviolet irradiation^70,71^. In fact, colour itself is not necessarily an important determinant of heat gain and heat loss^72^. The emittance of infrared radiation is hardly affected by colour^72^, while feather structure, plumage thickness and orientation towards the sun have a larger influence on heat transfer^69,73^. Also, heat transfer could be modified by behavioural adjustments like ptilomotor responses or postural changes^73–75^. For instance, Walsberg et al.^73^ showed how both light- and dark-plumaged pigeons *Columba livia* diminished their radiative heat loads when they made use of ptiloerection, which increased plumage thermal resistance by about 50%. Storks use ptiloerection and other postural adjustments^76^ which could contribute to diminish radiative heat load gain through their plumage. This might partly explain the absence of differences in the use of urohidrosis between darker and lighter species.

Likewise, latitude was not associated with the use of urohidrosis across species. This gives no support for the idea that tropical species likely rely more on urohidrosis than temperate species to dissipate heat^34^. Nevertheless, our species-specific analyses gave mixed support for this idea: a more pronounced use of urohidrosis near the tropics was found in the white stork and the jabiru, but the opposite was found in the painted stork and the greater adjutant. The white stork winters in the tropics and breeds in temperate latitudes, while the others occupy tropical and subtropical latitudes throughout the year^33^. Therefore, the effect of latitude on urohidrosis in the latter ones could be obscured, with urohidrosis use being associated with local environmental conditions rather than with latitude.

In contrast to our prediction, body mass did not emerge as significant predictor of urohidrosis use, although tarsus length had a marginally significant and positive effect (see Supplementary Table S2). We predicted that larger species (higher body mass and longer legs) would use urohidrosis more frequently due to their larger body water reserves and potentially larger thermal windows. However, all stork species usually have regular access to waterbodies^33^ and thus water should not be a strong limiting factor for maintaining their water balance, as reported for dessert passerines^46^. On the other hand, the marginal positive effect of tarsus length support the idea that species with longer legs use urohidrosis relatively more often to increase heat loss. Although tarsus length varies widely in the studied species (from 121 to 308 mm), its relative length (i.e. tarsus length to height ratio) was rather similar across species (from 3.98 to 6.44). Thus, the relative surface available to dissipate heat through contact of excreta and leg’s skin is quite similar across storks. This might explain why tarsus length was not a strong determinant of urohidrosis use.

Furthermore, our data indicates that urohidrosis is a well conserved behaviour across all stork species. This behaviour had also been reported in species from closely related phylogenetic groups like New World Vultures (Cathartidae), as well as in unrelated ones like boobies and gannets (Sulidae), suggesting it may be polyphyletic. Notably, species of these groups generally breed and/or forage in open landscapes where heat gain is typically high^41,43,45^. They also have relatively large legs that contribute significantly to the total uninsulated surface area of a bird. In addition to environmental pressures, large and highly vascularized legs—which are able to vasoconstrict and vasodilate in respond to hot or cold environmental conditions^34,35^—can be interpreted as preadaptations favouring the evolution of urohidrosis.

At the intraspecific level, we provided the first temperature thresholds of heat dissipation behaviours for large-bodied wading birds. Compared to other 50% thresholds of heat dissipation behaviours such as panting (from 33.9 to 46.1 $^\circ$C)^4,15^ or wing drooping (from 35.3 to 44.6 $^\circ$C)^4^ in small-sized desert birds, the environmental temperatures at which 50% of birds presented urohidrosis were generally lower (27.7 to 33.5 $^\circ$C). These differences could be explained by the fact that urohidrosis is probably cheaper (in terms of metabolic cost and water loss) than panting. In various groups of mammals, the increased output of saliva to provide water for evaporation from the respiratory track in response to heat stress is also utilized for evaporative cooling through saliva-spreading or licking^36,39,77^. Excess production of saliva, droppings (including cloacal evaporation,^78^) and other fluid secretions (e.g., diluted nasal mucous,^32^) could be seen as cheap means for heat loss by evaporative cooling, especially in birds since they lack sweat glands. This, combined with higher thermal inertia of larger birds, could favour the use of urohidrosis at lower temperatures in order to postpone the onset of physiological responses (e.g. panting, facultative hyperthermia) and/or maximize its cooling capacity.

The pigmentation of legs could play a signaling role besides their function as thermal windows. If so, urohidrosis could potentially result in a trade-off between thermoregulation and social signaling, as hypothesized for New World vultures^79^. This could be particularly crucial during mating and breeding which usually coincide with the highest annual temperatures. As in other birds, the colour of some storks’ legs is pigmented based and might act as a honest signal of mate fitness^80,81^. However, the potential influence of urohidrosis on signaling remains unexplored.

There are several methodological issues that may have impacted the results, although we do not think that they compromise the main inferences. Although scientifically curated repositories such as Macaulay Library are increasingly used to address numerous questions in conservation biology, ecology and evolution (e.g.^82–84^) we are aware of their limitations. First, we treated urohidrosis as a ‘point event’ behaviour but we could not ascertain from media files the exact moment in which it occurred. Therefore, we could not match urohidrosis events to hourly estimates of microclimate but had to use daily averaged values instead. This might have resulted in the absence of influence of wind at intraspecific level, as this variable is probably the most changing variable due to local topography or plant cover. Second, our approach prevented the association of urohidrosis with other heat dissipation behaviours and postures (e.g. panting, wing drooping, ptiloerection;^76^) that have a defined duration and could be potentially ranked into a sequence that reflect different degrees of thermal load. Third, one might argue that the more pronounced use of urohidrosis in ‘open’ species could be explained by passive washing of urohidrosis marks in ‘wading’ storks during foraging. Following this reasoning, photographs or videos taken after wading or bathing would show storks with clean legs, missing potential urohidrosis events. However, observations made during late spring in a colony of breeding white storks in southern Spain (unpublished data) proves that urohidrosis marks are quite persistent and usually do not disappear completely after wading or bathing (see Fig. S2), lasting for a variable timespan, from some hours to some days (up to 3 days) (J. Cabello-Vergel personal observation). Nonetheless, legs get progressively cleaner after several wading events. Thus, we cannot completely rule out that our indirect approach—based on photographs and videos randomly taken by birdwatchers around the world—has underestimated urohidrosis across all species considered in this study, not only in ‘wading’ species.

Despite these caveats, our study supports the notion that urohidrosis is correlated with overheating and the foraging ecology of birds. Yet, the energetics associated with this thermoregulatory behaviour (including the potential effect of the higher reflectivity of the leg’s surface covered by white excreta) remains to be studied in wild birds. Although Kahl^34^ experimentally showed that the internal body temperature of wood storks equipped with thermistor sensing probes change when increasing or decreasing heat loss from the legs and feet, all attempts to induce urohidrosis in birds wired for temperature readings and harnessed failed. Assuming that 3-kg storks excrete 1–2 cc of urine on their legs about every minute during short periods of heat stress, and that the evaporation of 1 g of water at 40 °C uses 575 cal, a sizable amount of heat may be dissipated from unfeathered parts of the leg. With modern thermal imaging cameras, it would be possible to quantify the cooling efficiency of urohidrosis along the leg surface and its contribution to overall heat loss (along with other bare parts like the bill).

Finally, this study demonstrates how data accumulated in digital resources combined with novel methods for computing microclimate can provide insight into animal thermoregulation. Future studies on this and similar cooling mechanisms (including the wetting of body surfaces in terrestrial and marine vertebrates;^32,36,37^) should significantly contribute to a better understanding of the significance of behavioural thermoregulation in hot environments. We propose that this method could be developed as a proxy for investigating community-level response to heat stress, and that it would be particularly relevant to predict vulnerability to climate warming scenarios.

## Supplementary Information

Supplementary Information.

## Data Availability

The raw data for our analysis (Supplementary Information Material S2) will be deposited in the Dryad Digital Repository upon acceptance.
