# Supplementary material for: Urohidrosis as an overlooked cooling mechanism in long-legged birds
Source: Sci Rep. 2021 Oct 8;11:20018. doi: 10.1038/s41598-021-99296-8 (PMC8501033; doi:10.1038/s41598-021-99296-8)
Supplement: Supplementary file 1 — Supplementary Information. [file 41598_2021_99296_MOESM1_ESM.pdf]

SUPPLEMENTARY MATERIAL S1

Urohidrosis as an overlooked cooling mechanism in long-legged birds

Julián Cabello-Vergel, Andrea Soriano-Redondo, Auxiliadora Villegas, José A. Masero, Juan M. Sánchez Guzmán and Jorge S. Gutiérrez

MATERIAL AND METHODS

Urohidrosis data collection

We looked for urohidrosis across all extant stork photographs and video recordings contained in Macaulay Library (https://www.macaulaylibrary.org; Cornel Lab of Ornithology). After discard those files in which urohidrosis could not be determined with confidence (those in which legs were not well visible those with low sharpness), we analyzed a total of 6,112 files. The number of files varied considerably across species: African openbill (n = 234), Asian openbill (n = 395), jabiru (n = 202), saddle-billed stork (n = 285), black-necked stork (n = 184), marabou (n = 576), lesser adjutant (n = 216), greater adjutant (n = 78), wood stork (n = 456), yellow-billed stork (n = 388), painted stork (n = 259), milky stork (n = 83), Abdim’s stork (n = 174), wooly-necked stork (n = 315), storm’s stork (n = 48), black stork (n = 446), maguari stork (n = 201), white stork (n = 1,534), and oriental stork (n = 38).

When several storks were present in the same file we determined the presence/absence of urohidrosis of each individual in which legs were well visible from top left to bottom right (see Fig. S1). Overall, we could extract 9,132 individual observations, distributed between species as follows: African openbill (n = 266), Asian openbill (n = 456), jabiru (n = 226), saddle-billed stork (n = 306), black-necked stork (n = 194), marabou (n = 708), lesser adjutant (n = 231), greater adjutant (n = 160), wood stork (n = 490), yellow-billed stork (n = 540), painted stork (n = 314), milky stork (n = 111), Abdim’s stork (n = 235), wooly-necked stork (n = 334), storm’s stork (n = 48),

black stork (n = 538), maguari stork (n = 210), white stork (n = 3,721) and oriental stork (n = 44).

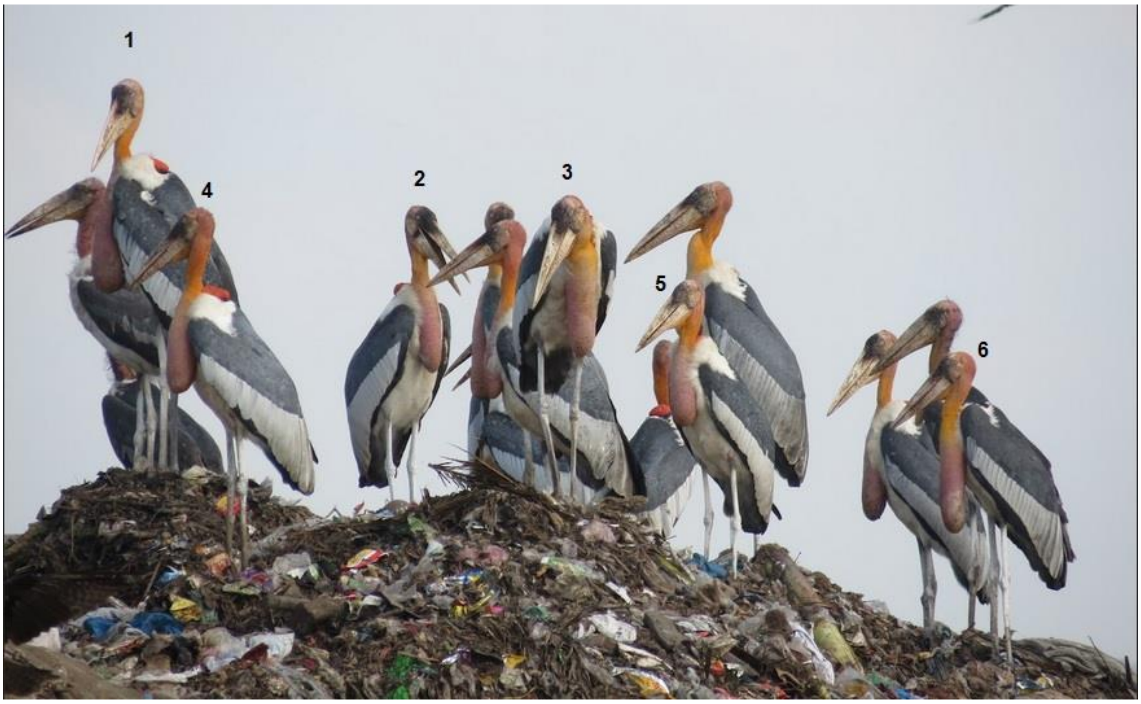

Figure S1. Example of urohidrosis data collection from a greater adjutant file employed in this study (photograph by Robin Welsh/Macaulay Library at the Cornell Lab of Ornithology, ML146223751). Numbers show the way in which urihodrosis scan in eac individual was performed.

During 2021 late spring we performed a study on thermoregulatory behaviour in a white stork's breeding colony placed near to Guadiana River (Mérida, Extremadura). During fieldwork we observed several events of urohidrosis when ambient temperature was high (>30 ºC). As previously demonstrated, this behaviour is intentional, with storks a) approaching their tibias to the cloaca or b) pointing the cloaca towards the legs in order to assure that the excreta hit their limbs. Once urohidrosis occurred, the white residue marks remained in the legs of known individuals for a variable timespan (from hours to up to 3 days), even after events of wading or bathing. When birds wade in the river in order to take food and/or water to their chicks, urohidrosis marks diluted to some extent and thus faded, thought they did not disappear completely (Fig. S2).

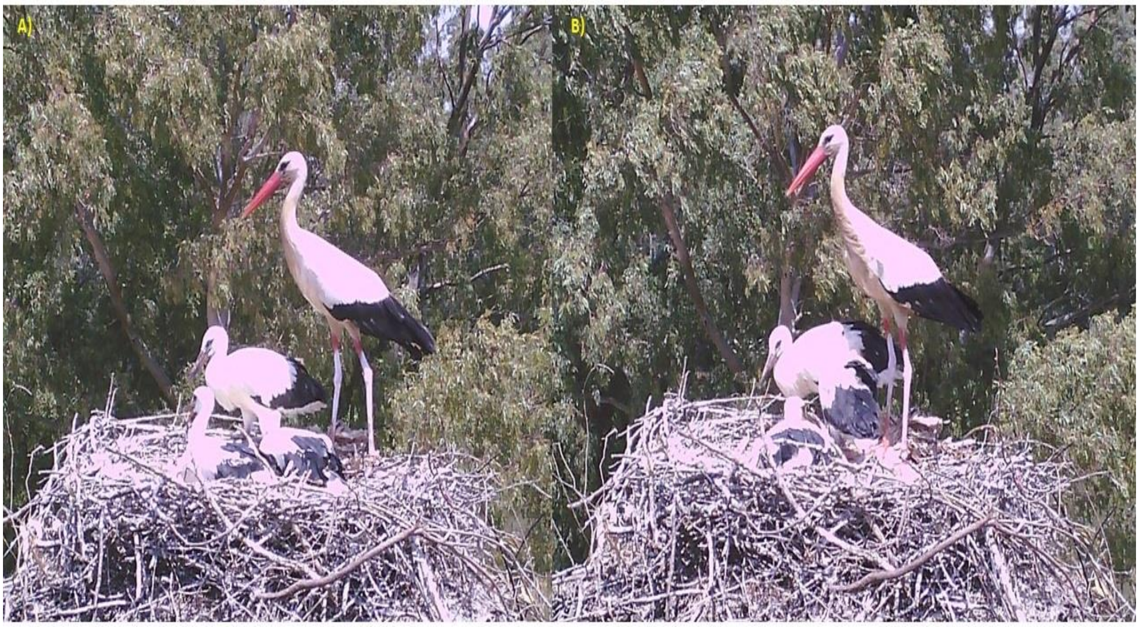

Figure S2. Urohidrosis residue marks can persist after wading, as illustrated by a comparison between photographs taken before a stork waded in the river (A) and after that single wading event (B). Photographs taken by the authors (J. C.-V.).

Microclimatic and latitudinal data

From the selected media files we could obtain microclimatic and latitudinal gradients representative of the natural distribution ranges for the majority of the species. That was not the case for storm's and milky storks, in which the low number of observations impeded our ability to obtain a representative gradient of temperature, radiation, wind speed, humidity and/or latitude values (see Table S1).

**Species**	**Temperature**	**Radiation**	**Wind speed**	**Humidity**	**Latitude**
Anastomus lamelligerus	6.80 – 43.14	0.07 - 3.40	0.38 - 6.72	0.002 – 0.022	0.05 - 27.77
Anastomus oscitans	7.37 – 44.31	0.10 - 2.13	0.26 - 8.09	0.003 – 0.026	1.00 - 31.70
Ciconia nigra	-0.78 – 45.28	0.00 - 3.46	0.31 - 9.39	0.001 – 0.029	0.02 - 59.19
Ciconia abdimii	17.43 – 39.43	0.16 - 2.42	0.48 - 7.24	0.004 – 0.023	0.02 - 29.68
Ciconia episcopus	2.93 – 43.98	0.13 - 2.39	0.23 - 10.85	0.002 – 0.026	0.07 - 32.57
Ciconia stormii	25.11 – 32.56	0.23 - 2.04	0.38 - 2.61	0.016 – 0.023	1.00 - 5.69
Ciconia maguari	13.41 – 38.64	0.13 - 2.47	0.88 - 10.53	0.003 – 0.026	1.00 - 40.81
Ciconia ciconia	-6.62 – 46.26	0.18 - 2.77	0.48 - 11.86	0.001 – 0.021	0.02 - 61.85
Ciconia boyciana	-2.99 – 29.25	0.15 - 1.78	1.00 - 12.25	0.001 – 0.022	1.00 - 40.13
Epphipiorhynchus asiaticus	2.55 – 42.95	0.30 - 2.41	0.60 - 9.96	0.002 – 0.023	1.00 - 30.93
Epphipiorhynchus senegalensis	18.04 – 39.27	0.38 - 2.36	0.70 - 7.44	0.003 – 0.022	0.02 - 28.39
Jabiru mycteria	17.18 – 39.67	0.13 - 2.31	0.57 - 7.70	0.005 – 0.025	1.00 - 30.87
Leptoptilos javanicus	5.20 – 43.97	0.12 - 2.38	0.23 - 7.65	0.003 – 0,027	1.00 - 28.49
Leptoptilos dubius	5.22 – 33.49	0.38 - 2.04	0.23 - 5.78	0.006 – 0.022	1.00 - 26.99
Leptoptilos crumenifer	17.38 – 41.11	0.00 - 3.40	0.27 - 6.48	0.002 – 0.017	0.00 - 29.60
Mycteria americana	10.62 – 39.70	0.03 - 2.50	0.37 - 13.11	0.004 – 0.024	0.69 - 41.99
Mycteria cinerea	25.04 – 30.93	0.14 - 1.92	0.28 - 4.66	0.014 – 0.025	1.00 - 14.90
Mycteria ibis	16.13 – 45.51	0.00 - 3.39	0.27 - 6.41	0.002 – 0.021	0.12 - 32.81
Mycteria leucocephala	16.76 – 43.91	0.10 - 2.14	0.40 - 8.09	0.002 – 0.027	1.00 - 31.39

Table S1. Temperature (ºC), radiation (MJ m^{-2} h^{-1}), wind speed (m s^{-1}), humidity (kg kg^{-1}), and latitude (as absolute) range collected for each species considered in this study.

RESULTS

Interspecific comparisons

Table S2. Posterior means of predictors from MCMC interspecific model (with 95% CI). Significant explanatory predictors are in bold.

	posterior Means	Lower CI	Upper CI	Effective sample	pMCMC
Intercept	- 6.364	-13.037	0.772	1,607.1	0.057
**Temperature**	**1.321**	**1.091**	**1.591**	**756.3**	**<5*e^{-04}**
**Radiation**	**0.884**	**0.665**	**1.097**	**736.4**	**<5*e^{-04}**
**Wind speed**	**- 0.294**	**- 0.468**	**- 0.134**	**1,688.2**	**0.001**
**Humidity**	**1.213**	**0.978**	**1.474**	**693.3**	**<5*e^{-04}**
Latitude	- 0.082	- 0.289	0.124	1,824.9	0.433
Body mass	- 2.430	- 6.097	1.023	1,809.3	0.147
Tarsus length	2.982	- 0.091	6,644	1,960.0	0.076
Plumage score	0.0345	- 1.516	1.606	1813.6	0.963
**Foraging:Open**	**7.441**	**1.618**	**13.804**	**1713.3**	**0.016**
Foraging:Wading	- 0.102	- 4.005	3.673	1960.0	0.941

Intraspecific comparisons

Table S3. Full model-averaged coefficients (ΔAICc < 2) for each stork species considered in intraspecific analyses. Significant explanatory predictors are in bold.

Species	Predictor	Estimate	SE	Adj SE	z value	95% CI
African openbill	**Intercept**	**-11.422**	**2.962**	**2.972**	**3.844**	**-17.246, -5.598**
	Temperature	0.136	0.149	0.149	0.907	-0.137, 0.185
	Radiation	0.049	0.937	0.941	0.052	-0.634, 0.646
	Wind speed	-0.115	0.646	0.649	0.177	-0.464, 0.436
	Humidity	0.378	0.902	0.906	0.417	-0.640, 0.739
	Latitude	-0.049	0.086	0.086	0.096	-0.060, 0.058
Asian openbill	**Intercept**	**-10.791**	**1.755**	**1.759**	**6.136**	**-14.238, -7.344**
	Temperature	0.058	0.106	0.106	0.542	-0.079, 0.095
	Radiation	0.116	1.045	1.047	0.110	-0.702, 0.730
	Wind speed	-0.172	0.479	0.480	0.359	-0.378, 0.333
	Humidity	0.429	0.562	0.563	0.761	-0.477, 0,619
	Latitude	-0.006	0.079	0.080	0.937	-0.055, 0.053
Abdim's stork	**Intercept**	**-8.022**	**2.196**	**2.207**	**3.634**	**-12.349, -3.696**
	**Temperature**	**0.233**	**0.067**	**0.067**	**3.473**	**0.102, 0.365**
	Radiation	0.541	0.480	0.483	1.121	-0.573, 0.989
	Wind speed	0.169	0.180	0.180	0.938	-0.185, 0.268
	Humidity	-0.330	0.380	0.382	0.865	-0.538, 0.380
	Latitude	-0.001	0.028	0.028	0.037	-0.016, 0.016
woolly-necked stork	**Intercept**	**-3.791**	**0.889**	**0.892**	**4.250**	**-5.540, -2.043**
	**Temperature**	**0.129**	**0.031**	**0.031**	**4.221**	**0.069, 0.189**
	Radiation	-0.516	0.290	0.291	1.771	-1.037, 0.291
	Wind speed	0.039	0.084	0.084	0.466	-0.059, 0.069
	Humidity	0.152	0.150	0.150	1.015	-0.165, 0.254
	Latitude	0.006	0.014	0.014	0.438	-0.010, 0.012

white stork	**Intercept**	**-21.300**	**1.949**	**1.950**	**10.921**	**-25.123, -17.478**
	**Temperature**	**0.883**	**0.068**	**0.068**	**13.024**	**0.748, 1.015**
	Radiation	1.673	0.914	0.915	1.829	-0.973, 3.279
	Wind speed	-0.064	0.409	0.409	0.157	-0.369, 0.344
	**Humidity**	**4.263**	**0.421**	**0.421**	**10.117**	**3.437, 5.089**
	**Latitude**	**-0.119**	**0.027**	**0.027**	**4.424**	**-0.172, -0.066**
marabou stork	**Intercept**	**11.056**	**2.021**	**2.025**	**5.461**	**7.088, 15.024**
	Temperature	0.071	0.145	0.145	0.491	-0.107, 0.127
	Radiation	0.149	0.722	0.723	0.206	-0.495, 0.532
	Wind speed	0.127	0.666	0.667	0.190	-0.456, 0.488
	Humidity	-0.487	0.897	0.898	0.542	-0.817, 0.677
	Latitude	0.015	0.077	0.077	0.191	-0.052, 0.532
greater adjutant	**Intercept**	**-31.435**	**12.158**	**12.233**	**2.570**	**-55.412, -7.458**
	Temperature	0.355	0.353	0.355	1.000	-0.363, 0.526
	Radiation	0.594	2.479	2.498	0.238	-1.768, 1.930
	Wind speed	-0.041	1.132	1.141	0.036	-0.819, 0.808
	Humidity	0.480	1.271	1.281	0.374	-0.935, 1.072
	**Latitude**	**1.510**	**0.371**	**0.374**	**4.037**	**0.777, 2.243**
lesser adjutant	Intercept	-1.555	0.943	0.947	1.642	-3.410, 0.301
	Temperature	0.051	0.031	0.031	1.623	-0.031, 0.106
	Radiation	0.718	0.413	0.415	1.730	-0.411, 1.456
	Wind speed	0.207	0.163	0.163	1.270	-0.201, 0.381
	**Humidity**	**0.737**	**0.235**	**0.236**	**3.119**	**0.274, 1.201**
	Latitude	0.006	0.027	0.027	0.205	-0.015, 0.016
yellow-billed stork	**Intercept**	**-13.000**	**3.433**	**3.438**	**3.781**	**-19.737, -6.261**
	Temperature	0.174	0.107	0.108	1.620	-0.095, 0.371
	Radiation	-0.113	0.592	0.593	0.190	-0.421, 0.394
	Wind speed	-0.116	0.445	0.446	0.261	-0.328, 0.299
	Humidity	0.067	0.670	0.671	0.099	-0.461, 0.445
	Latitude	-0.001	0.053	0.053	0.019	-0.036, 0.036
painted stork	**Intercept**	**-3.929**	**1.357**	**1.362**	**2.884**	**-6.599, -1.258**
	**Temperature**	**0.090**	**0.039**	**0.039**	**2.275**	**0.012, 0.168**
	Radiation	0.092	0.339	0.340	0.271	-0.272, 0.305
	Wind speed	0.016	0.119	0.119	0.137	-0.095, 0.101
	Humidity	-0.089	0.206	0.207	0.432	-0.206, 0.172
	**Latitude**	**0.059**	**0.025**	**0.025**	**2.307**	**0.009, 0.109**
wood stork	**Intercept**	**-6.341**	**1.447**	**1.451**	**4.370**	**-9.184, -3.497**
	**Temperature**	**0.149**	**0.044**	**0.044**	**3.362**	**0.062, 0.235**
	**Radiation**	**1.086**	**0.340**	**0.341**	**3.191**	**0.419,1.754**
	Wind speed	-0.107	0.085	0.085	1.260	-0.193, 0.104
	Humidity	0.212	0.183	0.184	1.156	-0.219, 0.379
	Latitude	-0.019	0.018	0.018	1.101	-0.035, 0.021
jabiru	**Intercept**	**-4.009**	**1.620**	**1.629**	**2.462**	**-7.201, -0.817**
	**Temperature**	**0.201**	**0.057**	**0.058**	**3.475**	**0.088, 0.314**
	Radiation	-0.341	0.390	0.392	0.869	-0.536, 0.382
	Wind speed	0.082	0.109	0.110	0.747	-0.100, 0.134
	Humidity	0.030	0.224	0.225	0.135	-0.170, 0.180
	**Latitude**	**-0.133**	**0.039**	**0.039**	**3.436**	**-0.209, -0.057**
